# A compression method for DNA

**DOI:** 10.1371/journal.pone.0238220

**Published:** 2020-11-25

**Authors:** Shengwang Du, Junyi Li, Naizheng Bian

**Affiliations:** College of Computer Science and Electronic Engineering, Hunan University, Changsha, Hunan, China; University of Helsinki, FINLAND

## Abstract

The development of high-throughput sequencing technology has generated huge amounts DNA data. Many general compression algorithms are not ideal for compressing DNA data, such as the LZ77 algorithm. On the basis of Nour and Sharawi’s method,we propose a new, lossless and reference-free method to increase the compression performance. The original sequences are converted into eight intermediate files and six final files. Then, the LZ77 algorithm is used to compress the six final files. The results show that the compression time is decreased by 83% and the decompression time is decreased by 54% on average.The compression rate is almost the same as Nour and Sharawi’s method which is the fastest method so far. What’s more, our method has a wider range of application than Nour and Sharawi’s method. Compared to some very advanced compression tools at present, such as XM and FCM-Mx, the time for compression in our method is much smaller, on average decreasing the time by more than 90%.

## Introduction

The advent of high-throughput sequencing technology has led to a dramatic increase in the size of DNA data. How to efficiently store them has become a new challenge. Many algorithms are proposed to compress DNA data in decades.

The CTW+LZ algorithm [1] is proposed by Matsumoto et al. The main feature of the algorithm is to combine the LZ algorithm and the context tree weighting algorithm to achieve the compression of DNA data. The algorithm first compresses each character in the DNA data using the LZ algorithm and the CTW algorithm, and simultaneously calculates the compression ratio under the two algorithms. Next, the compression ratio is sorted, the better algorithm is used to compress the current character. However, the CTW+LZ algorithm is very time consuming, it takes 8 minutes to compress the 38Kb sequence, which seriously affects the practicality of the algorithm. The DNACompress algorithm proposed by Chen [2] compares the biological sequences, and uses the Pattern Hunter software [3] to compress the obtained exact repeats and approximate repeats, and then uses the LZ algorithm to recompress the compressed fragments. But for other inexact repeats and approximate repeats, the arithmetic coding method is used for compression. In general, although the compression rate of the algorithm has not been significantly improved, the compression time has been saved a lot. Later, the DNAPack algorithm [4] proposed by Behzadi uses different methods for different segments in the sequence. For the approximate repeats in the sequence, the Hamming distance is used for the residual part; for the non-repetitive fragments in the sequence, the combination of the CTW algorithm and the second-order arithmetic coding is used. Later, Srinivasa further improved the algorithm and proposed the DNADP algorithm [5]. Unlike the DNAPack algorithm, the algorithm uses dynamic programming techniques to encode non-repetitive fragments in the sequence. The experimental results show that the algorithm can achieve a higher compression ratio, but the compression time is expanded by about 20 times.

The GeNML algorithm proposed by Tabus and Korodi [6] uses a special normalized maximum likelihood discrete regression model [7] (NMLComp, characterized by low spatial complexity) as a key part of the algorithm. It uses coding blocks and combines with the alternative principle to achieve compression of DNA data. Specifically, for a precisely repeated and approximately repeated DNA segment, a coding block is used for encoding processing; for other irregular segments, a context-based coding method is used for encoding processing; and for some specific sequences, no encoding is performed. Experiments using the algorithm show that compared with other DNA data compression algorithms, the algorithm obtains a larger compression ratio at the expense of more compression time.

In recent years, researchers have proposed some DNA data compression algorithms based on MA(Memetic Algorithm) and PSO(Particle Swarm Optimization) [8, 9]. POMA algorithm is one of the earliest algorithms using optimization calculation method [10], it is characterized by the maximum possible optimization operation of approximate repetitive vector (ARV), and it once again improves the compression ratio of DNA data, and it also proves that the optimization algorithm is practicality on the compression of DNA data, however, the disadvantage of the algorithm is that it is still relatively time consuming. In general, the algorithm is only suitable for small-scale optimization. Therefore, the researchers have proposed the COMRAD [11] algorithm. This algorithm can be applied to large-scale optimization, which can further improve the compression ratio, but the compression time required by the algorithm is not reduced.

In 2017, Nour S. Bakr and Amr A. Sharawi proposed a compression method for bacterial DNA sequence [12], which is the fastest method so far, but the scope of application is narrow, it is only applied to the compression of bacterial DNA sequence.

## Nour and Sharawi’s method

The first compression phase of this method takes advantage of the characteristics of bacterial biological DNA, resulting in limited applications. The second compression phase compresses the obtained file using bzip2 algorithm.

The first compression phase divides the sequence and stores it in different files in the form of characters.

In the first step of the first compression phase, the first 1000 base characters are taken from original sequence as a sample, the frequencies of four base characters A, T, G, and C are calculated and then sorted them in descending order. The four characters are named as the first (x1), second (x2), third (x3), and fourth (x4) frequency characters, respectively. Then three binary files (f1,f2,f3) are created when the original DNA sequence is traversed: write 1 to f1 for x1, write 0 for the rest; ignore x1, write 1 to f2 for x2, and write 0 to f2 for x3 and x4; ignore x1 and x2, write 1 to f3 for x3, and write 0 to f3 for x4.

This first compression is based on the base distribution law of the bacterial biological DNA data, that is, in most of the bacterial DNA sequence, the frequency of x1 is greater than the sum of the frequencies of x3 and x4. For example, if the original DNA sequence is TTGAACGATAATCCGTATTTGAAAAAAATT, the frequencies of A, T, G, C are 13, 10, 4, 3. In this case, 13 is greater than 4 plus 3. The binary code lengths obtained in three files are 30 (thirteen 1 and seventeen 0), 17 (ten 1 and seven 0), and 7 (four 1 and three 0), respectively. Only 54 bits are required to store 30 base characters.

However, the above compression strategy is only applicable to bacterial DNA sequence with the above characteristics. For example, if part of the DNA sequence in a bacterial organism is TGGACCGATATCGTATTTGAAGGACCTT, then the frequencies of A, T, G, and C are 8, 9, 7, and 6, respectively. If the first step is applied, the binary lengths obtained in the three files are 30 (nine 1 and twenty one 0), 21 (eight 1 and thirteen 0) and 13 (seven 1 and six 0), so 64 bits are needed to store 30 base characters. Actually we only need 60 bits to store 30 characters without compression. Therefore, the scope of application is narrow.

### Our method based on LZ77 algorithm

Directly using the general compression algorithm LZ77 to compress DNA data is not very effective. So we do some processing on the DNA data before we use LZ77 algorithm to compress them.

**Fig 1:**
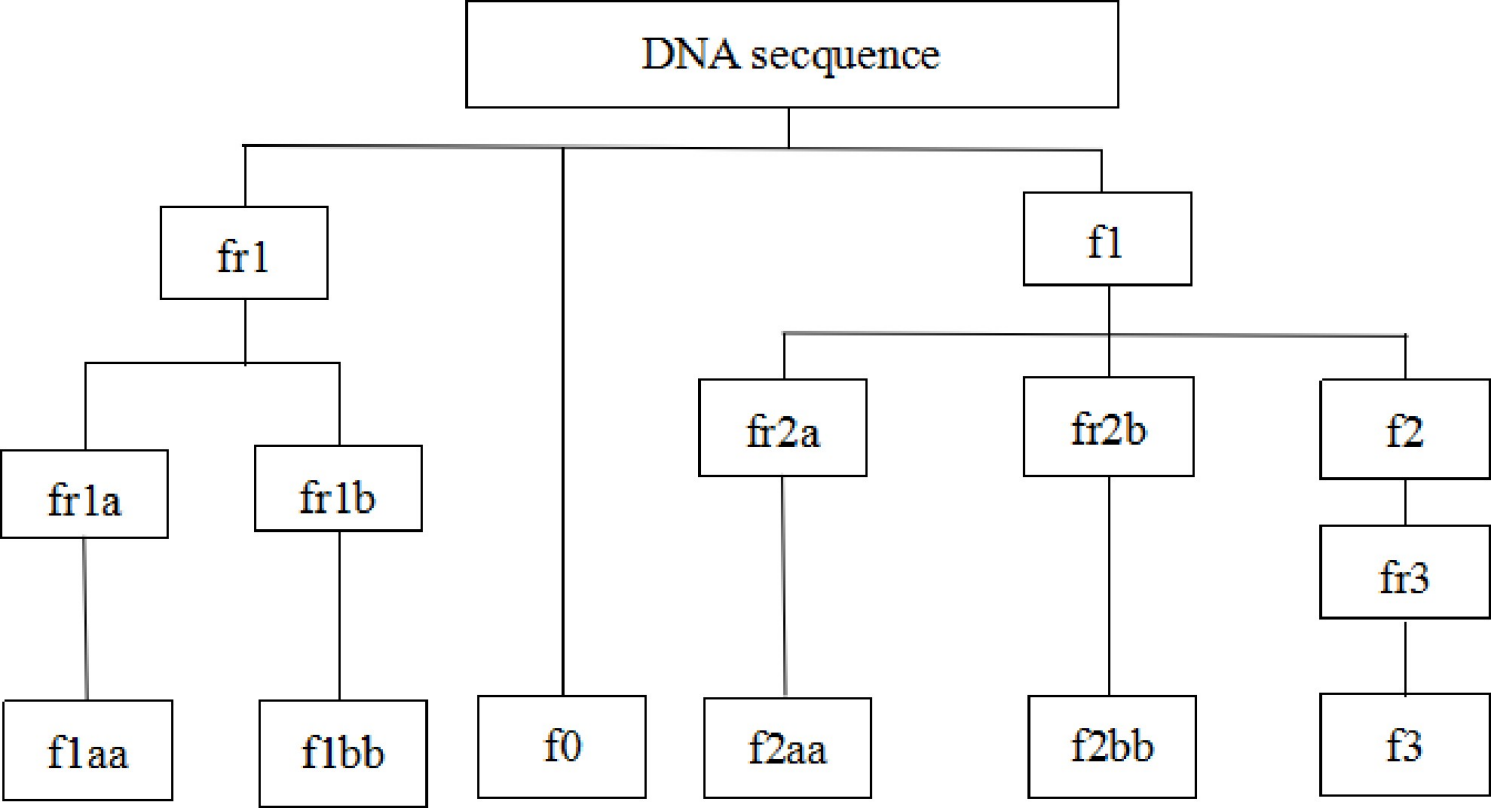
Files tree.

We proposed a compression method. The first compression is to convert the DNA data into ordinary characters and store them in different files. The second compression is to compress the ordinary character files using the LZ77 algorithm. The original sequences are converted into eight intermediate files and six final files. Fig 1 is the compression process and decompression process and the files tree.

#### (1) Compression

In the first phase of compression, we take six steps to generate six final character files and eight intermediate files. The step one, two, three and six refer to the method in literature [12].

Step one: Take the first 1000 characters in DNA secquence, the frequencies of the four base characters A, T, C, G are counted and sorted in descending order, temporarily named the first, second, third and fourth frequency character. Assume that a certain DNA data to be compressed is sorted into CATG after this step.
Step two: Traverse the DNA sequence, writing 1 to file fr1 when the first frequency character is encountered, writing 0 when other characters are encountered and adding the character to file f1. In this example, when C is encountered, 1 is written to file fr1, and when A, T, and G are encountered, 0 is written and the three characters A, T, and G are added to the file f1.
Step three: The largest file fr1 will be reduced in size. The operation is to traverse the content in the file fr1. If 0 is encountered and the number of consecutive occurrences is even, then even/2 zeros are written to file fr1a and 0 is written to file fr1b to represent an even number 0; If 0 is encountered and the number of consecutive occurrences is odd, then (odd +1)/2 zeros are written to file fr1a and 1 is written to file fr1b to represent an odd number 0; If 1 is encountered, writing to fr1a.
Step four (key step): The two pairs of characters in the file f1 are merged. Taking this as an example, the AA is converted to A and 0 is written to file fr2a (representing that this is the conversion of two identical characters) and A is added to the file f2; the AT is converted to G and 1 is written to file fr2a (representing that this is a conversion of two different characters), write 0 to file fr2b (the two characters are descended in frequency) and G is added to the file f2; the TA is converted to G and 1 is written to file fr2a (representing that this is a conversion of two different characters), write 1 to file fr2b (the two characters are in ascending order of frequency) and G is added to the file f2; The same treatments are encountered to TT, GG, TG, GT, AG, and GA.
Step five: Traverse the file f2, write 0, 10, and 11 to file fr3 for the second, third and fourth frequency characters in step one, respectively.
Step six: For file fr1a, fr1b, fr2a, fr2b, and fr3, each six binary group is converted into decimal and then added with 60, converting binary codes into corresponding ASCII codes, and write to f1aa, f1bb, f2aa, f2bb, f3, respectively. Then write the remaining 0 to 5 binary characters in the file f0 after the processing of the above five files and separated by #. At the same time write the base sort order obtained in the first step, separated by #, and then write the remaining 0 to 1 base character after the two characters of file f1 are merged. Finally the files fr1, fr1a, fr1b, fr2a, fr2b, fr3, f1, f2 are deleted.

In the second phase of compression, the LZ77 algorithm is used to compress the files f1aa, f1bb, f2aa, f2bb, f3 and f0.

Here is an example to illustrate our method.

The original sequence is:

TGGACCGTTAATCCTTTTTTGAAGGACCTT

The first phase of compression:

rank: TCAG

fr1: 100000011001001111110000000011

fr1a: 100011010111111000011

fr1b: 0000

f1: GGACCGAACCGAAGGACC

f2: GGAACCCCC

fr2a: 011001110

fr2b: 01101

fr3: 1111101000000

f1aa: _St

f1bb:

f2aa: U

f2bb:

f3: z\

f0: 011#0000#110#01101#0#TCAG#

The second phase of compression: six files (f1aa, f1bb, f2aa, f2bb, f3 and f0) are compressed by the LZ77 algorithm.

#### (2) Decompression

1. Use the LZ77 algorithm to decompress files (f1aa, f1bb, f2aa, f2bb, f3 and f0).
2. Process each file:
  Step one: Read the data in f0, break into tokens with delimiter #, save tokens to an array, a1.
  Step two: Read the data in f1aa, f1bb, f2aa, f2bb and f3, convert each character into number, subtract by 60, and then convert it into binary and write it to file fr1a, fr1b, fr2a, fr2b, fr3, respectively. Then get the first, second, third, fourth, fifth element from a1 and append them to fr1a, fr1b, fr2a, fr2b, fr3, respectively.
  Step three: Take the sixth element from a1, restore f2 from fr3. 11, 10, 0 are converted to three different base characters, respectively.
  Step four: Traverse the files f2, fr2a and fr2b, based on the sixth element in a1, restore the file f1, and add the seventh element in a1 to f1. The index of i1 is 0, and the index of i2 is 0. First, take one character and one number from f2 and fr2a where the index of the character and the number are equal to i1. If the number is 0, the character is restored to two identical characters, and then the value of the i1 is added 1, then continue to traverse; If the number is 1, then take one number from fr2b whose index value is equal to i2, if the number is 1, restore the original character to two other characters (in ascending order by frequency), if it is 0, then restore the original character to the other two characters (in descending order of frequency), and increase the values of i1 and i2 by 1 respectively to continue the traversal.
  Step five: Traverse the files fr1a and fr1b, restore fr1. In this step the largest binary file fr1 will be restored using both fr1a and fr1b files. Read a number from fr1a every time, if the number is 1, 1 is writted to binary file fr1. Else if the number is 0, continue to read and count the number of zeros until we get the number 1, then one number from the fr1b is read to show whether the number of zeros is odd or even. In the case of even number of zeros, the number of zeros is doubled and written to binary file fr1. In the case of odd number of zeros, the number of zeros is doubled and decreased by one and written to file fr1.
  Step six: Restore the original DNA sequence based on f1 and fr1.

For the same example in compression, the decompression process is as follows:

In the first phase of decompression, all files are extracted.

In the second phase of decompression, we restore fr1, fr1a, fr1b, fr2a, fr2b, fr3, f1, f2 and original DNA sequence.

a1: [011, 0000, 110, 01101, 0, TCAG,]

fr1a: 100011010111111000011

fr1b: 0000

fr2a: 011001110

fr2b: 01101

fr3: 1111101000000

f2: GGAACCCCC

f1: GGACCGAACCGAAGGACC

fr1: 100000011001001111110000000011

Restored original DNA sequence:

TGGACCGTTAATCCTTTTTTGAAGGACCTT.

## Results and discussion

We selected ten genomes with different lengths (1~15 M) from the NCBI(National Center of Biotechnology Information) database as a test data set, all data comes from “http://www.ncbi.nlm.nih.gov”, the data are collected by myself. They are tested on compression ratio, compression time and decompression time. The methods are applied on same machine(AMD A8-5550M APU with Radeon(tm) HD Graphics 2.10GHz CPU and 4.00GB of RAM) and the following is the experimental results. Table 1 are the datasets. The compression ratio is calculated as (uncompressed size-compressed size)/uncompressed size.

**Table 1:** The datasets.

Accession Number	Number of Bases
NC_017526	2682626
NC_002942	3397754
NZ_CP015934	3453407
NZ_CP015935	3409361
NZ_CP015938	3359444
NC_013929	10148695
NC_014318	1036715
NC_013595	10341314
NC_013131	10467782
NC_010162	13033779

Table 2 is the compression ratio, compression time and decompression time of our method and the fastest method.

**Table 2:** The results between our method and NSM.

SM	NSM			OM		
	CR	CT	DCT	CR	CT	DCT
NC_017526	75.35	21.227	10.109	75.00	6.004	5.311
NC_002942	75.41	29.772	12.612	75.02	5.351	4.947
NZ_CP015934	75.41	28.130	12.543	75.05	5.985	5.073
NZ_CP015935	75.40	28.507	13.264	75.02	5.529	5.733
NZ_CP015938	75.42	23.882	11.726	75.07	5.133	5.060
NC_013929	76.43	67.131	44.774	75.17	9.018	17.929
NC_014318	76.42	63.687	33.048	75.15	9.870	15.742
NC_013595	76.35	63.695	34.395	75.17	11.217	14.430
NC_013131	76.22	64.265	34.553	75.06	8.987	18.589
NC_010162	76.28	79.472	50.957	75.06	12.564	25.590
Average	75.87	46.977	25.798	75.08	7.966	11.840

SM: Sequence name.

NSM: Nour and Sharawi’ s method.

OM: Our method.

CR: compression ratio(%).

CT: compression time(s).

DCT: decompression time(s).

Table 3 is the compression ratio, compression time of our method and the two advanced compression tools.

**Table 3:** Compression benchmarks for state-of-the-art pure genomic compression tools.

SM	XM		FCM-Mx		OM	
	CR	CT	CR	CT	CR	CT
NC_017526	77.45	41.227	76.12	35.102	75.00	6.004
NC_002942	77.41	49.772	76.15	41.722	75.02	5.351
NZ_CP015934	77.41	48.130	76.39	39.603	75.05	5.985
NZ_CP015935	77.40	48.507	76.44	39.541	75.02	5.529
NZ_CP015938	77.42	43.882	76.21	35.796	75.07	5.133
NC_013929	77.43	107.131	76.33	99.305	75.17	9.018
NC_014318	77.42	103.687	76.67	95.850	75.15	9.870
NC_013595	77.35	103.695	76.15	95.208	75.17	11.217
NC_013131	77.22	114.265	76.13	103.209	75.06	8.987
NC_010162	77.28	139.472	76.17	124.167	75.06	12.564
Average	77.34	79.977	76.28	70.950	75.08	7.966

SM: Sequence name.

OM: Our method.

CR: compression ratio(%).

CT: compression time(s).

According to the experiment results, we can see that although our method has a slight reduction in the compression ratio, it reduces 83% of the compression time and 54% decompression time on average than Nour and Sharawi’ s method, which is the fastest method so far. What’s more, our method has a wider range of application than Nour and Sharawi’s method. Our compression method needs less compression time, the compression time is decreased by more than 90% on average than XM and FCM-Mx. The proposed method offers the best compression time and decompression when compared to all existing techniques.

## Conclusion

As the amount of DNA data continues to grow, we believe that he LZ77 algorithm, will play a key role in DNA data compression due to their simplicity and applicability.

Directly using general compression algorithms are not ideal for compressing DNA data. So it is necessary to perform some processing on the DNA data before using the general compression algorithm. The compression ratio was also improved compared with directly using LZ77 algorithm. The compression time is decreased by 83% and the decompression time is decreased by 54% on average and the compression ratio is almost the same compared with the fastest available method such as Nour and Sharawi’ s method and our method has a wider range of application. Therefore, our method has practical value for compression of DNA data.
